# Transmetallation Versus β-Hydride Elimination: The Role of 1,4-Benzoquinone in Chelation-Controlled Arylation Reactions with Arylboronic Acids

**DOI:** 10.1002/chem.201102678

**Published:** 2012-02-28

**Authors:** Christian Sköld, Jonatan Kleimark, Alejandro Trejos, Luke R Odell, Sten O Nilsson Lill, Per-Ola Norrby, Mats Larhed

**Affiliations:** [a] Dr.C. Sköld, A. Trejos, Dr. L. R. Odell, Prof. M. Larhed Organic Pharmaceutical Chemistry, Department of Medicinal Chemistry, Uppsala University,BMC, P.O. Box 574, SE-751 23 Uppsala (Sweden), Fax: (+46) 18-4714474; [b] J. Kleimark, Dr. S. O. Nilsson Lill, Prof. P.-O. Norrby Department of Chemistry, University of Gothenburg,Kemigården 4, 8076, SE-412 96 Göteborg (Sweden)

**Keywords:** arylation, C–C coupling, density functional calculations, elimination, palladium, transmetallation

## Abstract

**Abstract**The formation of an atypical, saturated, diarylated, Heck/Suzuki, domino product produced under oxidative Heck reaction conditions, employing arylboronic acids and a chelating vinyl ether, has been investigated by DFT calculations. The calculations highlight the crucial role of 1,4-benzoquinone (BQ) in the reaction. In addition to its role as an oxidant of palladium, which is necessary to complete the catalytic cycle, this electron-deficient alkene opens up a low-energy reaction pathway from the post-insertion σ-alkyl complex. The association of BQ lowers the free-energy barrier for transmetallation of the σ-alkyl complex to create a pathway that is energetically lower than the oxidative Heck reaction pathway. Furthermore, the calculations showed that the reaction is made viable by BQ-mediated reductive elimination and leads to the saturated diarylated product.

## Introduction

The Mizoroki–Heck reaction^1^ is a convenient and versatile method for the formation of carbon–carbon bonds through the vinylation or arylation of alkenes.^2^ The reaction is performed by Pd^0^ catalysis in which the active Pd^II^–vinyl or –aryl intermediate is produced by oxidative addition of aryl halides or pseudo-halides. Oxidative addition is followed by migratory insertion of the alkene to produce a σ-alkyl complex and β-hydride elimination from this complex leads to the unsaturated product. Finally, the catalytic cycle is completed from the hydridopalladium species by regenerating Pd^0^ by reductive elimination in the presence of a base. The Pd^II^-mediated version of the reaction with an organoboron compound to form the crucial Pd^II^–vinyl intermediate by transmetallation was reported by Dieck and Heck in 1975.^3^ Because hydridopalladium is still formed in the terminal step of the reaction, it was initially performed by using stoichiometric amounts of Pd(OAc)_2_. More recent developments have rendered the reaction catalytic by the re-oxidation of Pd^0^ to Pd^II^ and today the oxidative Heck reaction is an established and useful variant of the vinylic substitution reaction.^4^

**Scheme 1:**
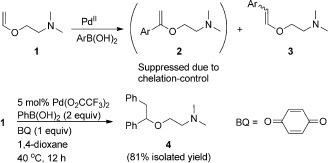
Scheme 1

One strategy to achieve regio- and stereoselectivity in the Mizoroki–Heck reaction has been by chelation-control, exploiting an auxiliary group to achieve a pseudo-intramolecular reaction pathway.2b, 5 By using the nitrogen-based chelating vinyl ether **1** (Scheme 1), Hallberg and co-workers developed a highly regioselective terminal substitution reaction.5a Whilst investigating a Pd^II^ catalytic protocol for the reaction (Scheme 1), employing arylboronic acids as Pd^II^–aryl precursors, we found that the unprecedented, saturated, diarylated product **4** was formed under certain reaction conditions (Scheme 1).^6^ Subsequently, efficient protocols, which employ both electron-rich and -poor arylboronic acids, have been developed.^6^, ^7^

**Scheme 2:**
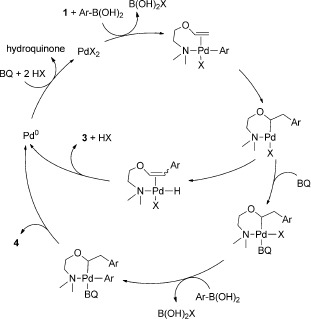
Hypothetic catalytic cycle for the production of 4 from 1 and an arylboronic acid (outer circle). Simplified catalytic cycle for the oxidative Heck reaction producing 3 is also shown (top half).

Chelation was shown to be vital for producing the saturated diarylated product because non-chelating alkyl vinyl ethers only yielded the Heck product. Interestingly, the choice of re-oxidant was also shown to be essential for the reaction outcome as only reactions with 1,4-benzoquinone (BQ) as re-oxidant gave this atypical product. The formation of the saturated diarylated product **4** is consistent with the interception of the σ-alkyl complex formed after migratory insertion by a second transmetallation and finally product formation by reductive elimination, in essence the latter part of the Suzuki–Miyaura reaction (Scheme 2). However, such a reaction pathway would require suppression of the competing β-hydride elimination from the σ-alkyl complex. Suppression of this process has been achieved by allylic stabilisation of the σ-alkyl complex in the similar diarylation of conjugated terminal alkenes by using organostannanes as the aryl source.^8^ Allylic stabilisation is not possible with **1** and some other mechanism must operate to suppress the β-hydride elimination. This intrigued us and we decided to further investigate this reaction. Thus, the aim of this study was to investigate the novel reaction pathway from **1** to **4** through the use of DFT calculations with a focus on elucidating the crucial role of BQ in the reaction. The reaction can be seen as a combination of the oxidative Heck reaction and the Suzuki–Miyaura reaction, for which an extensive list of theoretical investigations is available that have provided a profound mechanistic insight into these processes as well as the Mizoroki–Heck reaction.4c, 9, 10

## Results and Discussion

**Figure 1:**
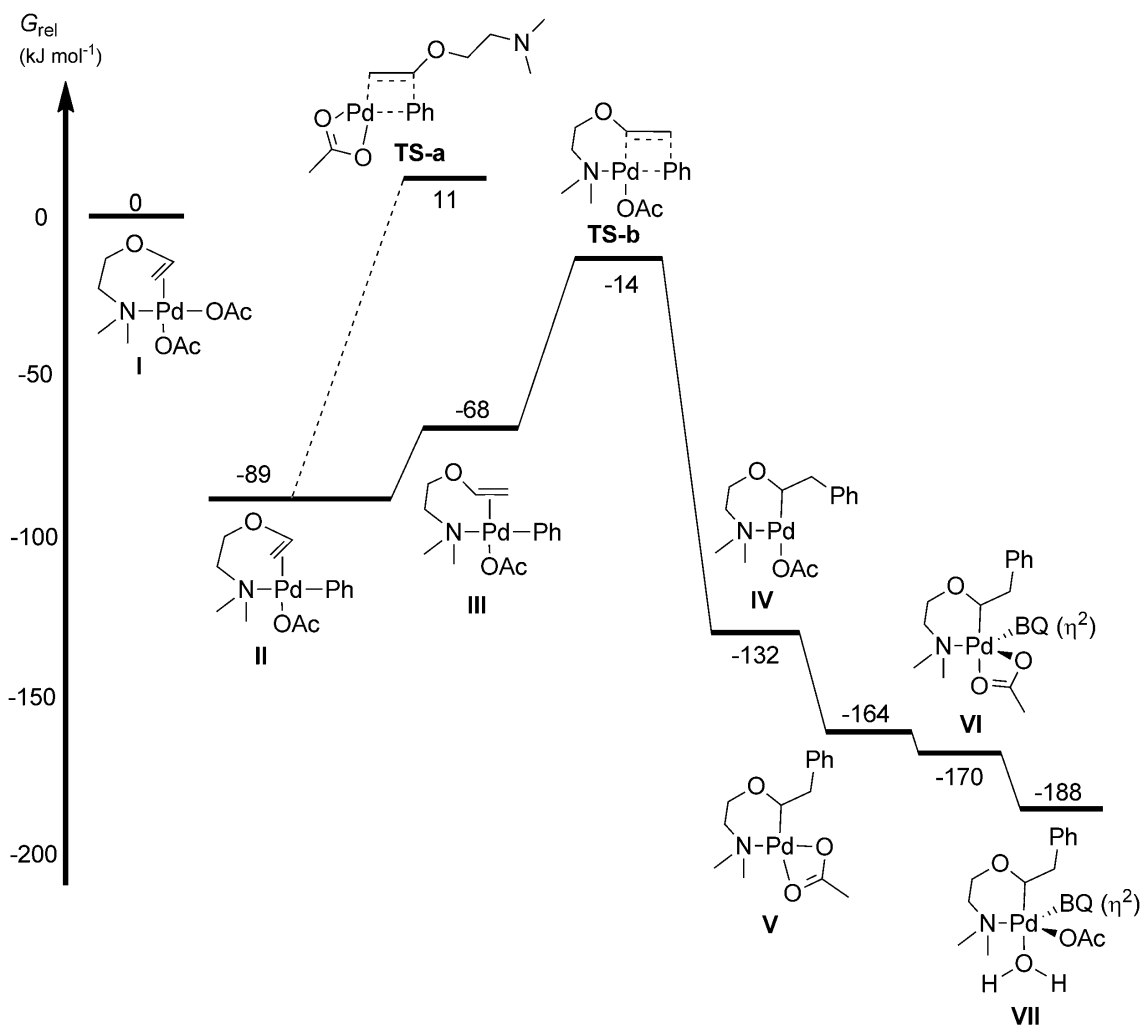
Free-energy profile for the migratory insertion, with energies relative to starting point I.

This investigation was focused on the reaction pathway following the first transmetallation step because the resulting Pd^II^–Ph complex will be formed regardless of the subsequent reaction path. Only neutral complexes were considered, because no additives promoting the cationic pathway (e.g., bidentate ligands) were present, and the reaction was performed in a non-polar solvent (dioxane). Potential bridged dimers of the complexes were excluded from the computational study. The computationally simple Pd(OAc)_2_ was used in the calculations and this is an experimentally functional Pd^II^ source, although the preparative optimisation revealed trifluoroacetate as the optimal Pd^II^ source.^6^, ^7^ Unless otherwise stated the B3LYP-D3 free energies presented herein are given relative to Pd(OAc)_2_ associated to **1** (complex **I**, Figure 1) because this complex was considered to be a suitable starting point for the reaction. A dashed line in the free-energy profiles indicates that the specific pathway for the connection (e.g., reactant to transition state) was not investigated.

**Migratory insertion**: Migratory insertions leading to both internal and terminal phenylation were computationally investigated, forming the σ-alkyl complexes, which after subsequent β-hydride elimination render **2** or **3**, respectively. The transition state (TS) for internal phenylation (**TS-a**) was found to be 25 kJ mol^−1^ higher in energy than the TS for terminal phenylation (**TS-b**). The free-energy profile is presented in Figure 1 and is in agreement with the experimental outcome of the reaction when the oxidative Heck product is formed, given that when chelation from the alkene is feasible, that is, no strongly coordinating bidentate ligand is present in the reaction mixture, the terminal product is formed with a **3**/**2** selectivity of >50:1.^6^ This is equivalent to the selectivity obtained with the Pd^0^-catalysed Mizoroki–Heck reaction.5a, 11 Accordingly, the pathway leading to internal phenylation was not further investigated. After migratory insertion, the free energy of the σ-alkyl complex is lowered when the acetate counterion changes binding mode from mono- to bidentate (**IV** to **V**, Figure 1).

**Figure 2:**
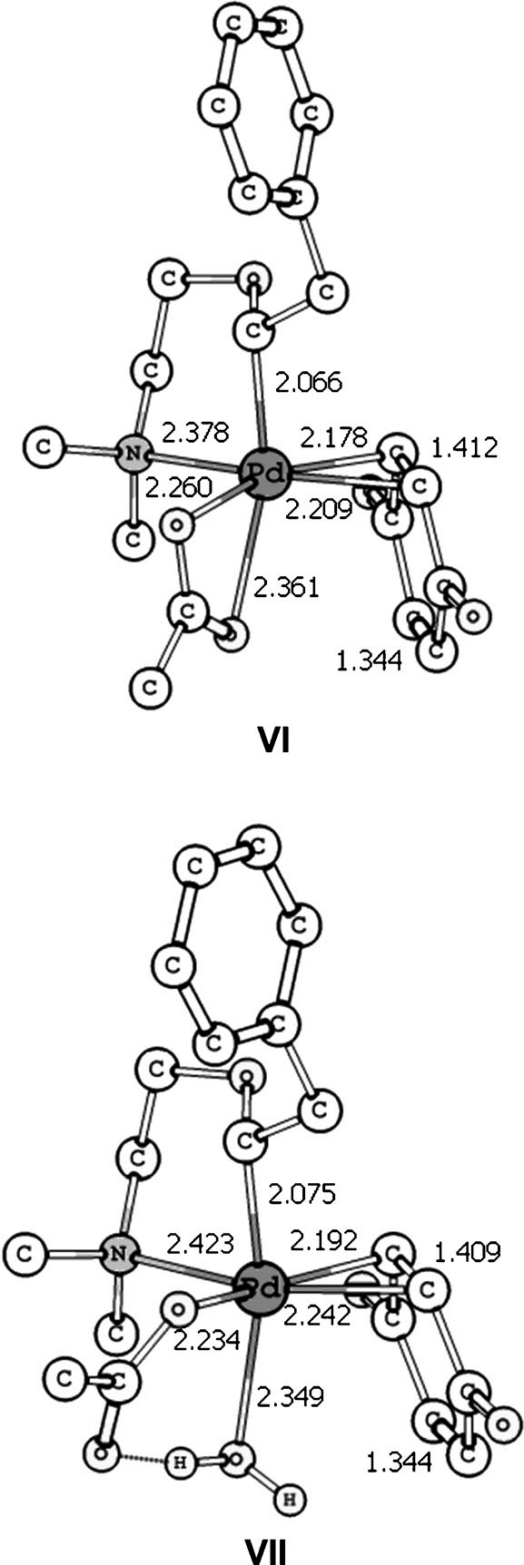
Optimised geometries of complexes VI and VII with bond lengths (Å) between palladium and coordinated atoms and BQ C–C double bonds. Hydrogen atoms have been omitted for clarity, except for the water molecule.

**η^2^-BQ association**: The post-insertion complex **V** is a square-planar Pd^II^ complex without any empty acceptor orbitals available for bonding. Thus, it will not coordinate additional dative ligands. However, in this study we found that complex **V** could associate with a η^2^-BQ ligand^12^ to give complex **VI** with an approximate trigonal-bipyramidal geometry (Figure 2). This finding led us to investigate the nature of the Pd–BQ bond in more detail. A natural bond orbital (NBO) analysis^13^ revealed a very strong donation–back-donation situation in the second-order perturbation analysis. In fact, the energy contribution calculated for the donation from BQ to palladium was similar in magnitude to that from each acetate oxygen atom, and about twice that of the donation (dative bond) from the nitrogen lone pair. In addition, the back donation from a filled d orbital on palladium to the BQ π* orbital approximately equalled the donation contribution. Thus, the complex has a strong contribution from a metallacyclopropane resonance form with a formal oxidation state of +4 on palladium. This should not be confused with a pure Pd^IV^ complex, but the contribution is still strong enough to allow palladium to achieve an octahedral geometry in which the two coordinating BQ carbon atoms occupy two coordination positions. A similar geometry has been reported in a crystal structure of a formal Pd^II^ complex with maleic anhydride as the electron-deficient alkene.^14^ The octahedral geometry is more clearly seen in the even more stable complex **VII** (Figure 2) in which one water molecule has replaced one acetate oxygen. The contribution from the metallacyclopropane form can also be seen in the lengthening of the coordinating BQ carbon–carbon bond (by ca. 0.07 Å), which clearly shows significant single-bond character, and in the pyramidalisation of the same carbon atoms (see the Supporting Information).

**β-Hydride elimination versus transmetallation**: After forming the σ-alkyl complex the following reaction steps are selectivity-determining: the oxidative Heck β-hydride elimination or the Suzuki–Miyaura transmetallation. Accordingly, these two different reaction pathways were considered from **VII**. For β-hydride elimination the transition states **TS-c**–**TS-e** in Table 1 were computationally investigated, forming (*Z*)-**3** with acetate interacting with the transferred hydride in the TS, (*E*)-**3** and (*Z*)-**3**, respectively. Their calculated energies are presented in Table 1. The lowest free-energy barrier for β-hydride elimination was found to be via **TS-e**, the path leading to (*Z*)-**3**. However, the difference in free energy for the pathway leading to the (*E*)-**3** was only 1 kJ mol^−1^, which is in accord with the poor diastereoselectivity observed when the oxidative Heck product is formed.^6^

**Table 1:** Comparison of the free energies of the investigated β-hydride-elimination TSs.

TS	Complex	Δ*G*^[a]^ [kJ mol^−1^]
**TS-c**	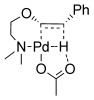	121
**TS-d**	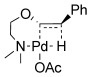	116
**TS-e**	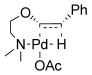	115

[a] Free-energy barrier, relative to the preceding stationary minima **VII**.

For transmetallation, pathways with the associated acetate to activate the boronic acid and pathways via Pd–OH complexes were investigated. The process of counterion exchange, association of the boronic acid and subsequent dihedral rotation was not calculated (i.e., from **VII** to the pre-transmetallation complex), because numerous pathways are possible and it is unlikely that the free-energy barriers for this process are higher than the competing β-hydride-elimination barrier of 115 kJ mol^−1^. One possible pathway is the acetate-assisted deprotonation of water in **VII** to form a Pd–OH intermediate, which has been shown to be the active intermediate in the Suzuki–Miyaura reaction.^15^ From this Pd–OH complex, the association of boronic acid and the dissociation of acetic acid would produce **VIII**. The free-energy barrier for the deprotonation of water coordinated in a Pd^II^ complex has been reported to be 25 kJ mol^−1^ in a computational study of the Wacker process,^16^ albeit by using a different calculation method and conditions, such as the solvent and ligands, used herein. The barrier for dissociation/association has been estimated to be diffusion limited with a value in the order of 20 kJ mol^−1^.^17^ Furthermore, in another computational investigation, focusing on transmetallation in the Suzuki–Miyaura reaction, it was found that the transmetallation TS is higher in energy than any of the complexes preceding the TS.10a Thus, it is reasonable to assume that in our study the TS for transmetallation is also the highest in energy for this part of the reaction.

The calculated energies of the different transmetallation TSs investigated are presented in Table 2. First, transmetallation reactions of complexes without BQ associated were calculated. By utilising the associated acetate to activate the boronic acid, the six- and four-atom transmetallation TSs (Table 2, **TS-f** and **TS-g**, respectively) were both calculated to be 6–12 kJ mol^−1^ higher in energy than the transmetallation via Pd–OH (Table 2, **TS-h**). Notably, a comparison of the lowest free-energy barriers to β-hydride elimination (**TS-e**) and transmetallation without BQ association (**TS-h**) showed that the latter was calculated to be preferred by 10 kJ mol^−1^. However, these values were calculated by using settings that reflect the optimised reaction conditions leading to **4**, and not the oxidative Heck product **3**, for which DMF has been used as solvent in the experimental protocol.^6^ Intriguingly, by changing the parameters to be suitable for DMF instead of dioxane in the solvent model the preferred reaction pathway was indeed calculated to be the β-hydride elimination (**TS-e**), which was 17 kJ mol^−1^ less than the energy of the competing transmetallation pathway when BQ was absent (**TS-h**). However, with polar solvents, a cationic Pd^II^-complex reaction pathway^11^ may need to be investigated; this is beyond the scope of this investigation.

**Table 2:** Comparison of the free energies of the postulated transmetallation TSs.

TS	Complex	Δ*G*^[a]^ [kJ mol^−1^]
**TS-f**	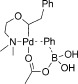	111
**TS-g**	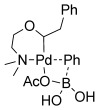	117
**TS-h**	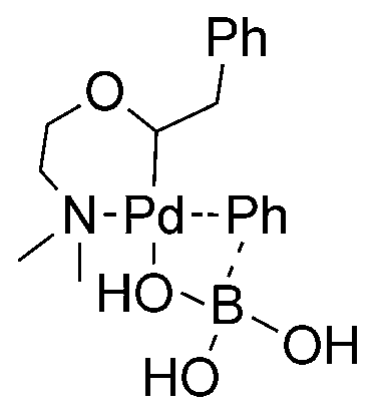	105
**TS-i**	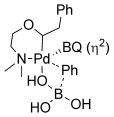	85^[b]^

[a] Free-energy barrier relative to the preceding stationary minima **VII**.

**Figure 3:**
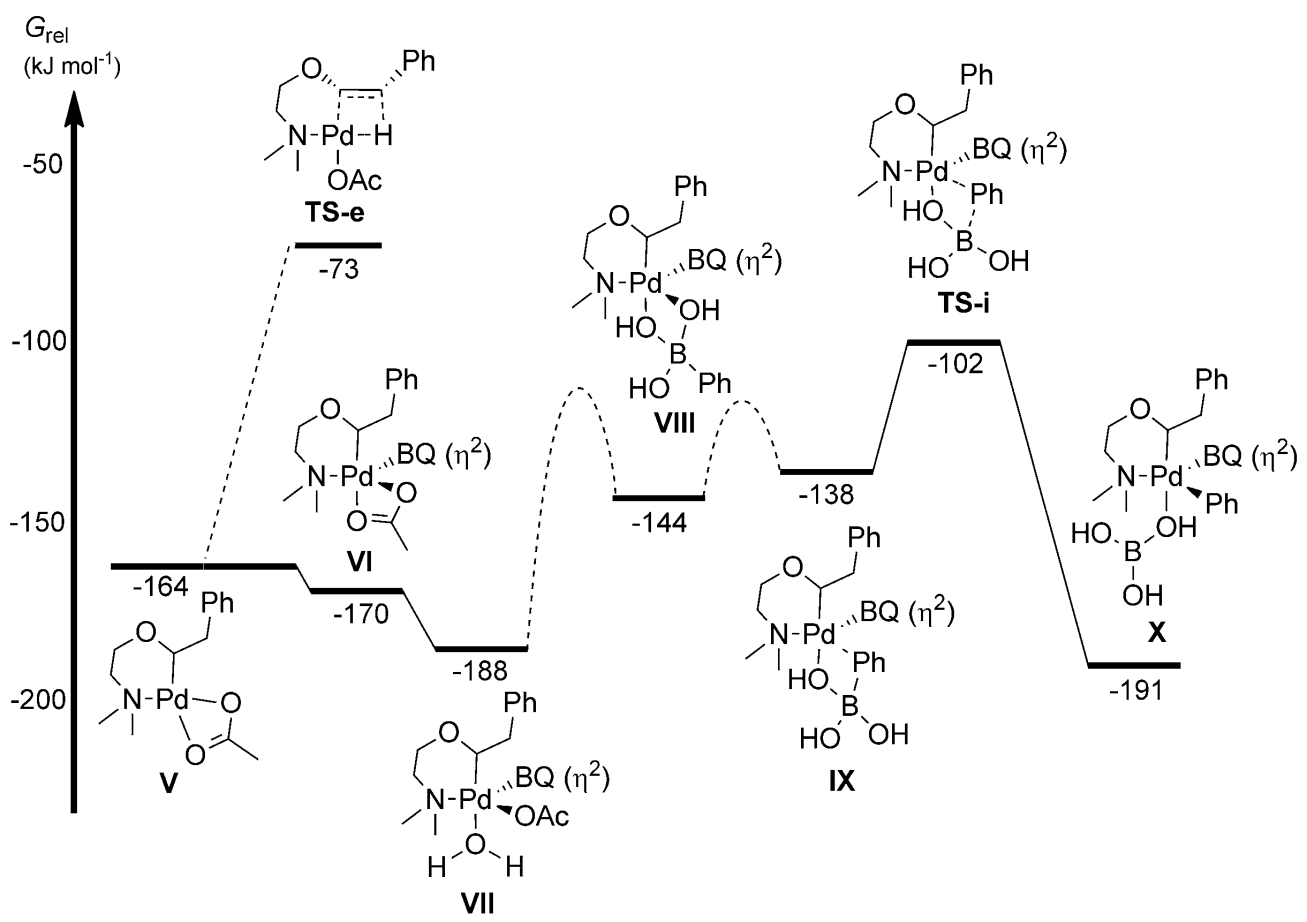
Free-energy profile of the lowest-found free-energy pathway for β-hydride elimination versus transmetallation.

[b] This value differs from the value of the barrier of 86 kJ mol^−1^ presented in Figures 3 and 6 due to rounding of the *G*_rel_ values.

When BQ was included in the transmetallation step the calculated free energy required was found to be 20 kJ mol^−1^ lower (Table 2, **TS-i**); this strongly suggests that BQ aids this transmetallation step. As stated above, BQ coordination to the σ-alkyl complex to form **VI** and **VII** results in the back donation of electrons to the electron-deficient BQ double bond, increasing the electrophilicity of Pd^II^. This should promote transmetallation and is thus in full agreement with the experimental outcome of the reaction in which a second transmetallation takes place before **4** is formed. Note that correction for dispersion had a profound effect on the free energies for these sterically congested complexes. The inclusion of dispersive interactions has been shown to have importance, not only for the binding energies of non-covalently bound hydrocarbons^18^ or biochemical compounds involving aromatic residues,^19^ but also for transition-metal–ligand binding energies^20^ or metal–metal bonds.^21^ Recently it has also been shown that dispersive interactions can have a large impact on activation energies. For example, Harvey and co-workers have shown this for oxidative-addition reactions that involve transition metals^17^ and Siegbahn et al. have similarly described the effect on enzymatic reactions that involve transition metals.20d We therefore anticipated that BQ coordination would have an effect on the activation barriers of the reactions involved in this study. At the outset of the study, it was not clear whether the effect would be stronger in the resting state, which would result in an increased activation barrier, or in the transition state, which would result in a lowering of the net activation barrier. Gratifyingly, it was found that inclusion of the correction for dispersion had a profound effect in reducing the free-energy activation barriers for the steps in which BQ was coordinated to palladium, whereas TSs lacking the coordination of BQ experienced a relative increase in the activation barrier. In particular, the relative energies for **TS-e** and **TS-i** were shifted when the dispersion correction was included. Thus, without dispersion correction β-hydride elimination would have been identified as the energetically preferred reaction pathway.

Because dispersion correction was vital for any conclusions to be made we also performed energy calculations in this step of the reaction by employing the M06 functional. Relative to the calculated energies obtained by employing B3LYP-D3, the energy difference between the competing TSs—**TS-e** and **TS-i**—was reduced from 30 to 14 kJ mol^−1^. Also, the stabilising effect of the coordination of BQ to the σ-alkyl complex was much less profound as the relative energies of **V**–**VII** were found to be within 2 kJ mol^−1^ by using the M06 functional. Nevertheless, it is clear that the M06 calculations also support transmetallation as the energetically preferred pathway rather than β-hydride elimination.

The effect on the relative free energies of **TS-e** and **TS-i** of changing the solvent was also investigated. By using parameters appropriate for DMF in the solvent model the calculated relative energies changed, which resulted in a barrier to β-hydride elimination that is 13 kJ mol^−1^ lower than that for transmetallation. This suggests that performing the reaction in DMF strongly hampers the production of **4** in favour of **3**, regardless of the addition of BQ to the reaction mixture. This is in line with the experimental results that showed that polar solvents increased the production of **3**.^6^, ^7^ However, an energy difference of 13 kJ mol^−1^ should result in the almost complete selectivity of **3**, which indicates an overestimation of the calculated difference in free energy. But as stated above, the possibility of a cationic Pd^II^ complex pathway should also be investigated before any final conclusion on the predictability of the calculations, regarding the effect from polar solvent, can be made.

A prerequisite for forming a Pd–OH complex that can activate PhB(OH)_2_ to give the calculated lowest-energy transmetallating complex **VIII** is that water is present in the reaction mixture. The experimental protocol^6^, ^7^ did not include water, but the reagents and solvent used in the protocol likely contained small amounts of water. To investigate the effect of water content under controlled reaction conditions, eight experiments were performed in which the concentration of water in the reaction was varied, starting from anhydrous conditions (Table 3).

**Table 3:** Yields of 4 produced in reactions with different amounts of water in the reaction mixture.^[a]^
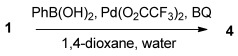

Amount water [equiv]	Yield^[b]^ [%]	Amount water [equiv]	Yield^[b]^ [%]
0	3	5	38
0.01	12	10	41
0.1	30	30	28
1	60	100	24

[a] Reagents and conditions: PhB(OH)_2_ (4 equiv), BQ (1.5 equiv), **1** (1 equiv), Pd(CF_3_COO)_2_ (0.05 equiv) and water (when added) in 1,4-dioxane (1.5 mL) at 40 °C for 24 h. [b] Determined by ^1^H NMR analysis of the crude product.

These experiments revealed that anhydrous reaction conditions were detrimental for the reaction, with **4** produced in only 3 % yield. With increased water content the yield of **4** increased up to the addition of one equivalent of water, after which the yield steadily decreased, presumably due to protodeboronation or oxidation of the phenylboronic acid.^22^ The maximum yield of **4** in the eight experiments was 60 % according to ^1^H NMR analysis of the crude product, which was lower than the previously reported 81 % isolated yield. However, because this investigation focused on the effect of water content in the reaction and not on the optimisation of yield, no attempts were made to increase the yield. One possible explanation for this discrepancy in yields could be that the optimal amount of water was not included in the series, possibly residing in the range of 0.1–5 equivalents of water.

Taken together these results support the hypothesis that transmetallation in the σ-alkyl complex is the energetically preferred pathway and proceeds starting from a Pd–OH complex, aided by the associated BQ. The free-energy profile of the low-energy competing pathway is presented in Figure 3.

**Reductive elimination**: After transmetallation, to produce intermediate complex **X**, reductive elimination would render the final product **4**. The free energies for the different reductive elimination pathways investigated are shown in Table 4. Without coordinated BQ, the free-energy barriers for the reductive elimination starting from **X** were high, 144 kJ mol^−1^ for the tetracoordinated complex **TS-j** employing dioxane as a ligand and 142 kJ mol^−1^ for the tricoordinated complex **TS-k**. The inclusion of BQ in the complexes had a large effect on the energy barrier, lowering it to 95 kJ mol^−1^ for the tricoordinated complex **TS-l** and 72 kJ mol^−1^ when the dimethylamino group was also coordinated, giving **TS-m**. The effect of BQ on the reductive elimination step was not surprising because the use of electron-deficient alkenes (π acids) as ligands to promote reductive elimination has been investigated and reported by others.^23^

**Table 4:** Comparison of the free energies of the reductive elimination TSs considered herein.

TS	Complex	Δ*G*^[a]^ [kJ mol^−1^]
**TS-j**	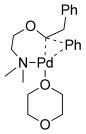	144
**TS-k**	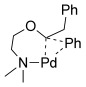	142
**TS-l**	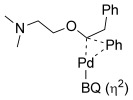	95
**TS-m**	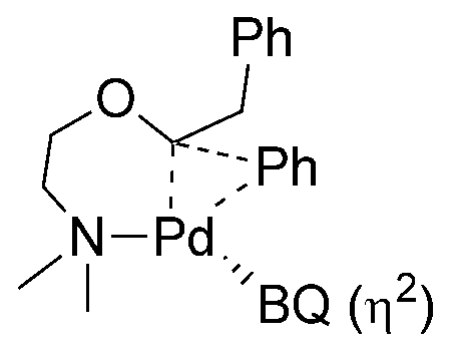	72

[a] Free-energy barrier relative to the preceding stationary minima **X**.

**Figure 4:**
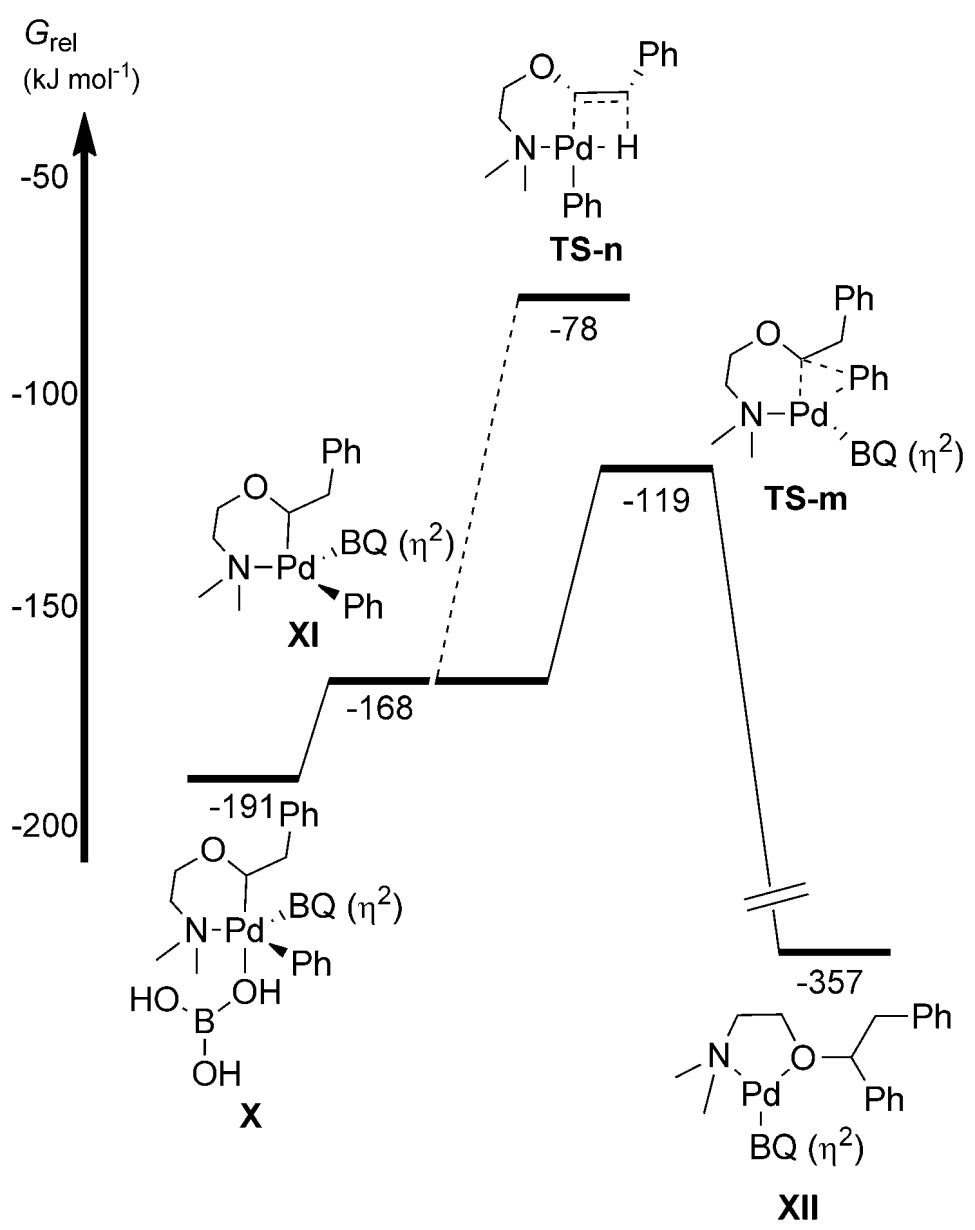
Free-energy profile of the lowest-found free-energy pathway for reductive elimination versus β-hydride elimination.

A possible path to the oxidative Heck product (*Z*)-**3** by β-hydride elimination starting from complex **X** was also investigated. The energy barrier for β-hydride elimination was calculated to be 112 kJ mol^−1^, well above the energy barrier of the BQ-mediated reductive elimination. Thus, the calculated energies support a significant reduction of the energy barrier for the reductive elimination with BQ as ligand, but excluding BQ from the reaction would inhibit the reductive elimination pathway because the energy barrier would be greater than that for the β-hydride elimination. The free-energy profiles for the competing reaction pathways are shown in Figure 4.

**Figure 5:**
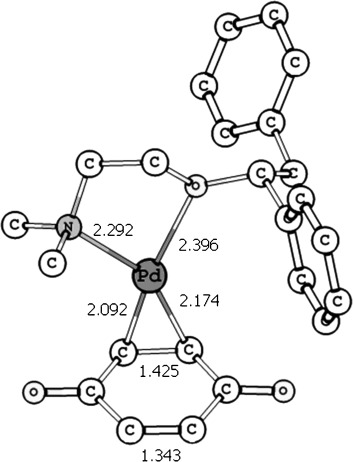
Optimised geometry of complex XII with bond lengths (Å) between palladium and coordinated atoms and BQ C–C double bonds. Hydrogen atoms have been omitted for clarity.

The product-forming reductive elimination step is highly exergonic, forming complex **XII** as depicted in Figures 4 and 5. This complex is a formal Pd^0^ complex with product **4** coordinated in a bidentate mode through its nitrogen and oxygen atoms and BQ coordinated through one of its electron-deficient double bonds. As in the case of **VI** and **VII**, the coordinated BQ also affects the geometry of **XII** resulting in a square-planar geometry, which is the preferred geometry of Pd^II^. This could once again be explained by the π-accepting properties of BQ. A crystal structure of a related square-planar complex with formal Pd^0^ coordinating 2,2′-bipyridine as a bidentate ligand and η^2^-BQ has been reported.^24^

**Scheme 3:**
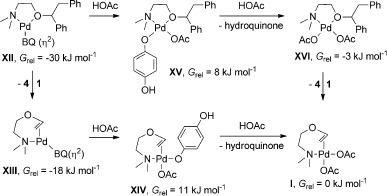
Two potential oxidation pathways from XII to the starting point I.

**Closing the catalytic cycle**: The TSs for palladium oxidation were not investigated, but two potential reaction pathways involving acetic acid as the proton source are presented in Scheme 3 for a more complete picture of the reaction leading to the starting point **I**. Although the TSs have not been investigated, the pathway involving the coordination of the product has lower-energy intermediates, but both pathways are viable.

**Figure 6:**
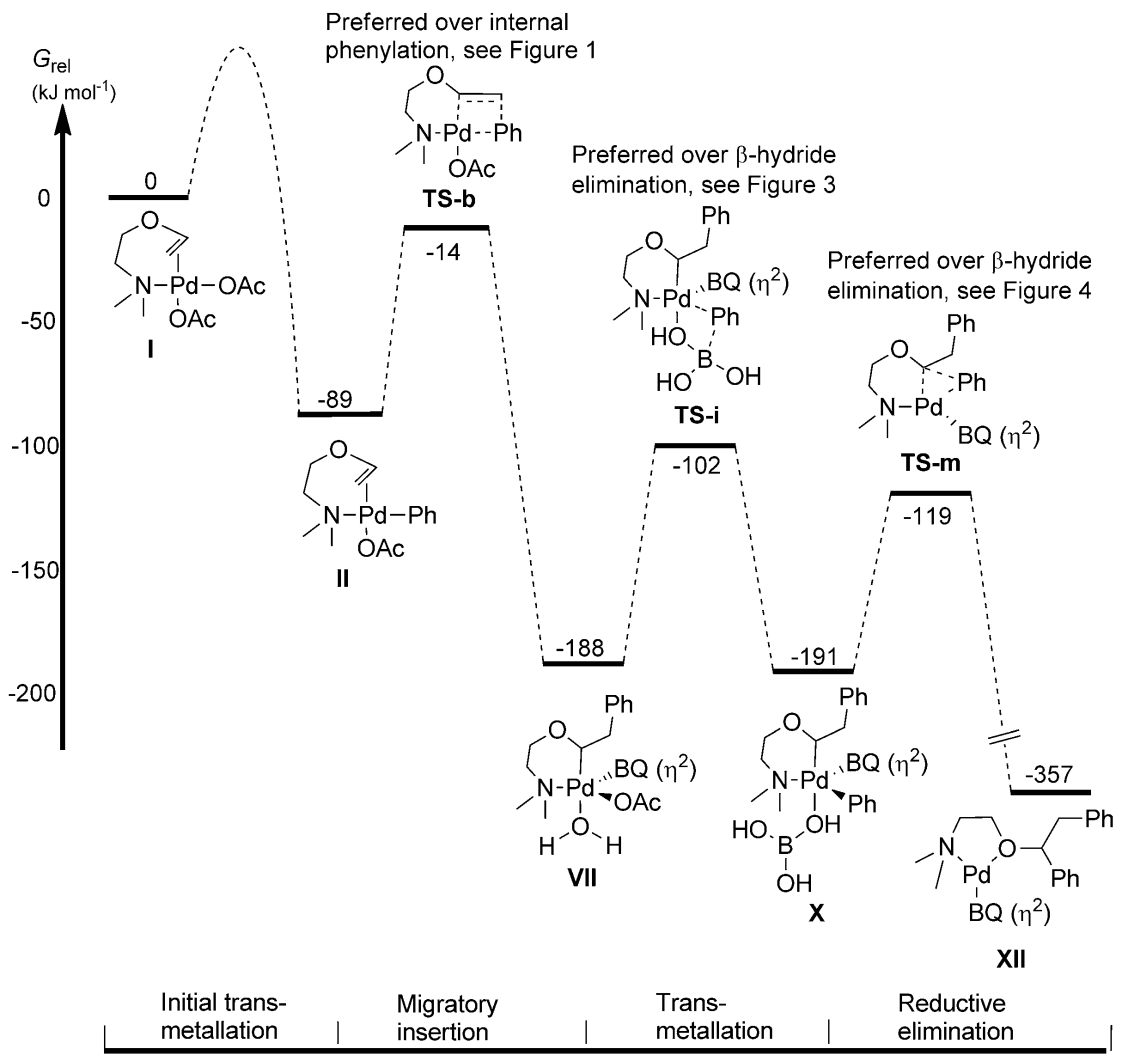
Free-energy profile of the complete reaction.

**Complete reaction**: After the initial transmetallation and migratory insertion, the calculations support the view that the association of BQ by the σ-alkyl complex leads to a low-energy pathway, both for the transmetallation and the formation of the final saturated diarylated product by reductive elimination, in essence confirming the catalytic cycle postulated for the reaction (Scheme 2). The validity of this conclusion is supported by the difference in free energy of 30 kJ mol^−1^ (or a lower, but significant, free-energy difference of 14 kJ mol^−1^ by employing M06) for β-hydride elimination compared with BQ-assisted transmetallation. This energy difference even allows for computational uncertainties and the calculations indeed predict the correct experimental outcome of the reaction. Furthermore, because the TSs of the competing pathways start from the same stationary minimum, the relative free energies of the TSs should be less affected by computational uncertainties. On the contrary, the absolute free energies of the reaction barriers are subject to uncertainties, because of the number of potential ligands in the reaction mixture that leads to a large number of possible complexes preceding the TSs. We believe that the most probable low-energy complexes have been included in the calculations. A summary of the preferred reaction pathway is presented in Figure 6. Of the reaction steps that were calculated, transmetallation has the highest free-energy barrier, but in accord with the statement above, it is difficult to conclude whether this is the rate-determining step. It is clear that both migratory insertion and reductive elimination are effectively irreversible as no subsequent step has a transition-state energy that is even close in energy. This is probably also true for the transmetallation step, but the difference with respect to the subsequent reductive elimination is lower, and therefore the conclusion less certain. However, the competing β-hydride elimination is high enough in energy to prevent entry to this path through the reversibility of the transmetallation step under the investigated reaction conditions.

## Conclusion

We have used DFT calculations to theoretically investigate a reaction in which an intermediate σ-alkyl complex of a chelation-controlled, oxidative Heck reaction is intercepted by transmetallation with an arylboronic acid followed by reductive elimination to form a saturated diarylated product. The calculated reaction pathway is in excellent agreement with the experimental outcome and the computational results show why 1,4-benzoquinone has an essential role in the reaction. In addition to its role as an oxidant of palladium, to complete the catalytic cycle, this electron-deficient alkene lowers the energy barrier for the reductive elimination, making the reaction viable. Furthermore, the calculations show that 1,4-benzoquinone also lowers the free-energy barrier for the transmetallation of the σ-alkyl complex, ensuring a low-energy pathway below that of the oxidative Heck reaction pathway.

## Experimental Section

**Computational details**: The calculations were performed by using Jaguar, version 7.6,^25^ with the B3LYP hybrid functional^26^ and the LACVP** basis set, which uses an effective core potential^27^ for palladium and 6-31G** for all other atoms. All geometries were optimised in the gas phase with a subsequent single-point energy calculation in the solution phase utilising the PBF solvation model^28^ with parameters suitable for dioxane (dielectric constant, *ε*=2.2 and probe radius=2.5719389) and DMF when relevant. Vibrational analyses were performed on the optimised geometries in the gas phase and the free energies of the geometries were calculated by adding the thermodynamic contribution at 298.15 K to the solution-phase energy. Dispersion correction was calculated for the gas-phase geometries by using the DFT-D3 program^29^ (version 2.0, rev. 1) and was added to obtain the final energies. For the calculations performed with the M06 hybrid functional^30^ and the LACVP** basis set, single-point energy calculations in the solution phase were performed on the optimised geometries obtained by the B3LYP calculations and the thermodynamic contribution at 298.15 K was added to obtain the final energies. The transition states in the lowest free-energy path on the potential energy surface were determined to be connected to their corresponding reactants and products by geometry optimisation in the forward and backward direction from the TS. All the TSs presented have exactly one imaginary frequency and the stationary minima have no imaginary frequencies.

**Water concentration investigation**: Anhydrous 1,4-dioxane was obtained from Sigma–Aldrich and used without further purification. The reaction vials were prepared in an atmosphere of N_2_(g) by using a N_2_(g)-flushed AtmosBag (Sigma–Aldrich). The 1,4-benzoquinone and phenylboronic acid used in the reaction were recrystallised and dried under vacuum prior to use. Pd(CF_3_COO)_2_ was obtained from Strem Chemicals and used as received. An 8 mL reaction vial was charged with phenylboronic acid (127.0 mg, 4 equiv), 1,4-benzoquinone (42.6 mg, 1.5 equiv), *N*,*N*-dimethyl-2-(vinyloxy)ethanamine (30.0 mg, 1 equiv) and anhydrous 1,4-dioxane (1.5 mL). The mixture was homogenised by stirring and then Pd(CF_3_COO)_2_ (4.3 mg, 0.05 equiv) and water (when added) were added to the reaction mixture, which was then heated in a metal heating block at 40 °C for 24 h. The mixture was then diluted with EtOAc (20 mL) and washed with NaOH (1 m, 3×15 mL). The aqueous phase was analysed by LC-MS to ensure no product could be detected. The organic phase was dried with K_2_CO_3_, filtered and concentrated. The crude material was then analysed by NMR spectroscopy (CDCl_3_) after the addition of a known amount of DMF, which served as an internal standard.
